# Sodium Thiosulfate Improves Intestinal and Hepatic Microcirculation Without Affecting Mitochondrial Function in Experimental Sepsis

**DOI:** 10.3389/fimmu.2021.671935

**Published:** 2021-06-07

**Authors:** Jan Schulz, Sandra Kramer, Yasin Kanatli, Anne Kuebart, Inge Bauer, Olaf Picker, Christian Vollmer, Richard Truse, Anna Herminghaus

**Affiliations:** Department of Anesthesiology, University Hospital Duesseldorf, Duesseldorf, Germany

**Keywords:** sodium thiosulfate, glibenclamide, microcirculation, mitochondria, gut, rat, sepsis

## Abstract

**Introduction:**

In the immunology of sepsis microcirculatory and mitochondrial dysfunction in the gastrointestinal system are important contributors to mortality. Hydrogen sulfide (H_2_S) optimizes gastrointestinal oxygen supply and mitochondrial respiration predominantly *via* K(ATP)-channels. Therefore, we tested the hypothesis that sodium thiosulfate (STS), an inducer of endogenous H_2_S, improves intestinal and hepatic microcirculation and mitochondrial function *via* K(ATP)-channels in sepsis.

**Methods:**

In 40 male Wistar rats colon ascendens stent peritonitis (CASP) surgery was performed to establish sepsis. Animals were randomized into 4 groups (1: STS 1 g • kg^-1^ i.p., 2: glibenclamide (GL) 5 mg • kg^-1^ i.p., 3: STS + GL, 4: vehicle (VE) i.p.). Treatment was given directly after CASP-surgery and 24 hours later. Microcirculatory oxygenation (µHBO_2_) and flow (µflow) of the colon and the liver were continuously recorded over 90 min using tissue reflectance spectrophotometry. Mitochondrial oxygen consumption in tissue homogenates was determined with respirometry. Statistic: two-way ANOVA + Dunnett´s and Tukey post - hoc test (microcirculation) and Kruskal-Wallis test + Dunn’s multiple comparison test (mitochondria). p < 0.05 was considered significant.

**Results:**

STS increased µHbO_2_ (colon: 90 min: + 10.4 ± 18.3%; liver: 90 min: + 5.8 ± 9.1%; p < 0.05 vs. baseline). Furthermore, STS ameliorated µflow (colon: 60 min: + 51.9 ± 71.1 aU; liver: 90 min: + 22.5 ± 20.0 aU; p < 0.05 vs. baseline). In both organs, µHbO_2_ and µflow were significantly higher after STS compared to VE. The combination of STS and GL increased colonic µHbO_2_ and µflow (µHbO_2_ 90 min: + 8.7 ± 11.5%; µflow: 90 min: + 41.8 ± 63.3 aU; p < 0.05 vs. baseline), with significantly higher values compared to VE. Liver µHbO_2_ and µflow did not change after STS and GL. GL alone did not change colonic or hepatic µHbO_2_ or µflow. Mitochondrial oxygen consumption and macrohemodynamic remained unaltered.

**Conclusion:**

The beneficial effect of STS on intestinal and hepatic microcirculatory oxygenation in sepsis seems to be mediated by an increased microcirculatory perfusion and not by mitochondrial respiratory or macrohemodynamic changes. Furthermore, the effect of STS on hepatic but not on intestinal microcirculation seems to be K(ATP)-channel-dependent.

## Introduction

Sepsis and the consecutive multiorgan dysfunction syndrome (MODS) are still a major burden in critical care medicine (1). To prevent MODS, it is of increasing clinical interest to maintain gastrointestinal as well as liver microcirculatory and mitochondrial function and thereby ensure adequate organ performance (2, 3). Insufficient blood supply might lead to gastrointestinal barrier failure with translocation of bacteria and toxins in the blood and lymph system, hypoxic hepatitis and a dysfunctional immune response thereby increasing patients’ mortality (4, 5).

In this context, hydrogen sulfide (H_2_S), a gaseous mediator, gained growing attraction in the therapy of microcirculatory and mitochondrial organ dysfunction (6). For example, exogenous H_2_S increased intestinal blood flow, reduced mesenteric ischemia and preserved LPS-induced organ injury and death in experimental animal models (7, 8). Furthermore, H_2_S can maintain mitochondrial function in sepsis as well as in ischemia-reperfusion models and reduce oxygen-consumption to protect cellular integrity (6, 9, 10). Thereby, the vasoactive and the mitochondrial effect of H_2_S seems to be K(ATP)-channel-dependent (11–13).

However, administering gaseous H_2_S in a clinical setting remains difficult. A simple technique to elevate H_2_S-levels is the application of sodium thiosulfate (STS). STS increases endogenic H_2_S concentration *via* enzymes, especially under ischemic conditions (14, 15). Thereby, STS can be administered intravenously and in the experimental setting also intraperitoneally and is standardly used in humans to treat calciphylaxis, cyanide intoxication as well as in the prevention of ototoxicity during cisplatin treatment (16–18). However, recent experimental studies have also shown that STS preserves mitochondrial function in ischemic hearts and improves survival in endotoxic mice, mainly mediated due to H_2_S (8, 19). Nevertheless, data for the gastrointestinal effect of STS under septic conditions are missing and it remains unclear if this potential effect of STS is also K(ATP)-channel-dependent.

Therefore, we conducted this randomized, placebo-controlled, blinded trial in septic rats to evaluate the effects of STS and glibenclamide on the intestinal as well as hepatic microcirculation and mitochondrial function.

## Materials and Methods

All parts of this study were performed in accordance with NIH guidelines for animal care and reported in accordance with the ARRIVE guidelines. Experiments started after approval from the local Animal Care and Use Committee (Landesamt für Natur, Umwelt und Verbraucherschutz, Recklinghausen, Germany, Az. Az. 84-02.04.2015.A538).

### Surgical Induction of Sepsis

**Figure 1:**
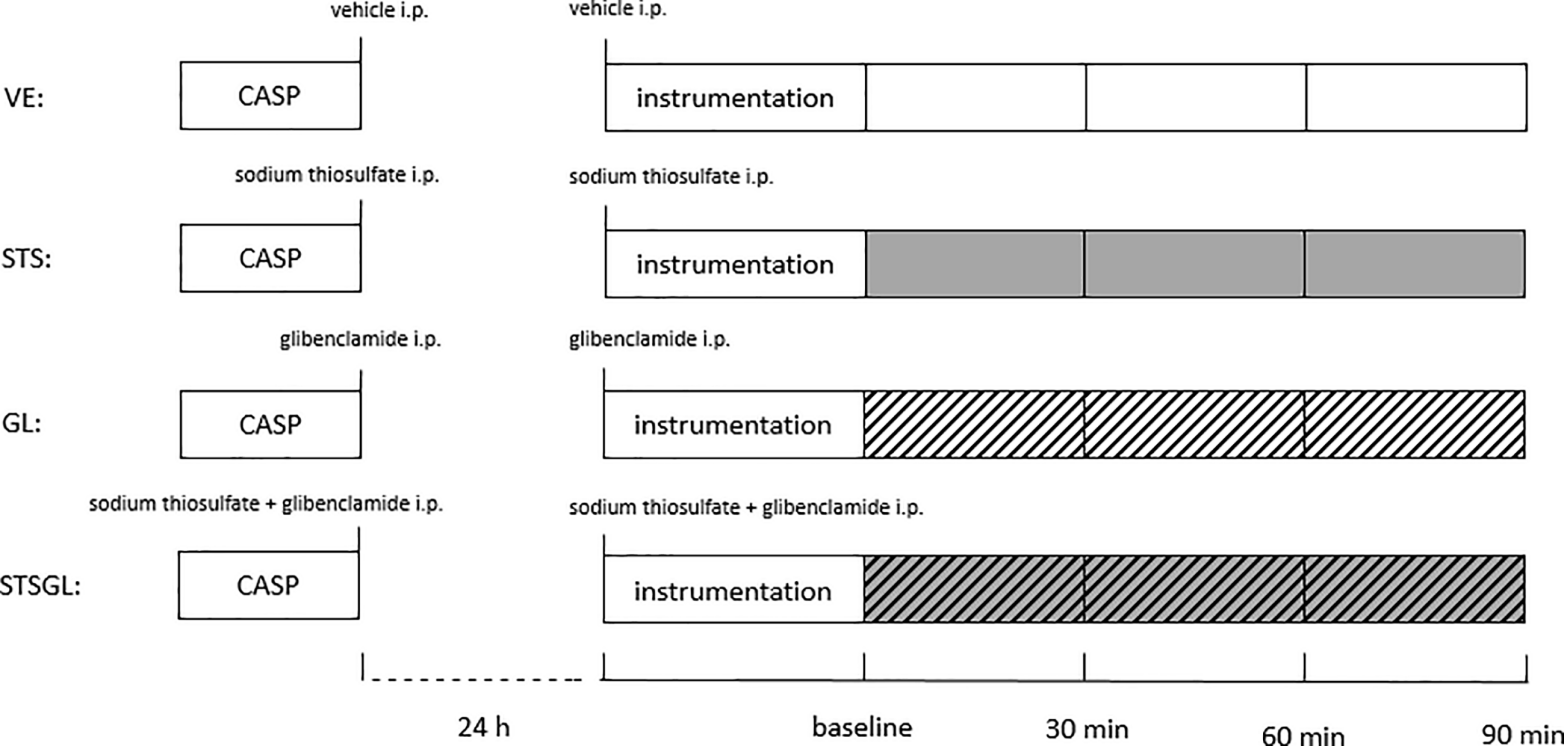
Experimental protocol. Colon ascendens stent peritonitis (CASP)-surgery was carried out 24 h before the experiment. Intraperitoneally treatment with vehicle (VE), sodium thiosulfate (STS), glibenclamide (GL) or sodium thiosulfate + glibenclamide (STSGL) was carried out after CASP surgery and directly before the experiment.

The animals were derived from the breeding facility of the Heinrich-Heine-University Duesseldorf. 48 male Wistar rats (320 - 380 g body weight) were randomly assigned to one of the 4 experimental groups (**Figure 1**). However, 8 animals died within 24 hours after induction of sepsis, so experiments were performed in 40 rats (n = 10). It is of note, that there was no statistically relevant difference between the groups concerning death within the first 24 hours. The experiments started at 8:00 a.m. in the research laboratory of the Heinrich-Heine-University Duesseldorf, Dept. of Anesthesiology. Colon ascendens stent peritonitis (CASP)-surgery was performed with two 16-gauge PVC to develop sepsis using an established protocol as described previously (20) (21). Our previous studies demonstrate this model to be adequate to induce moderate sepsis in contrast to irreversible septic shock models. Anesthesia was induced and maintained by sevoflurane (3.0-3.2 % end-expiratory concentration, F_i_O_2_ 0.5) and buprenorphine (0.05 mg • kg^-1^, s.c.). A 2 cm-long median laparotomy was performed, the colon was located and penetrated 1 cm distal to the ileocecal valve with two 16-gauge PVC (Vasofix safety, B. Braun Melsungen AG, Melsungen, Germany). The inner needles were withdrawn, allowing constant fecal leakage into the abdominal cavity to develop abdominal sepsis. The intestine was carefully returned and the abdominal wall was closed. Afterwards, animals received vehicle (1.75 ml crystalloid fluid (Jonosteril Fresenius Kabi, Bad Homburg, Germany) and 1 ml DMSO (Dimethyl sulfoxide D8418, Sigma-Aldrich, Taufkirchen, Germany)), sodium thiosulfate (1.0 g • kg^-1^, i.p., sodium thiosulfate (in Jonosteril) 217263, Sigma-Aldrich, Taufkirchen, Germany) and glibenclamide (5 mg • kg^-1^, i.p. (in DMSO), glybenclamide G0639, Sigma-Aldrich, Taufkirchen, Germany) or both. The same treatment was applied 24 h after sepsis induction. The investigator was blinded to the treatment.

After CASP-surgery, animals were kept individually in separate plastic cages at a 12-h light/dark cycle with free access to water and food under controlled temperature (24 ± 2°C) and humidity (50% ± 5%). Buprenorphine (0.05 mg • kg^-1^ s.c.) was applied at 8 h and 16 h after surgery. As described previously, animals were examined and scored every 6 h according to a defined protocol (Septic Rat Severity Score: SRSS) to determine the severity of sepsis and to monitor the animals with respect to their welfare (loss of body weight, appearance, spontaneous behavior, provoked behavior, breathing rate, expiratory breathing sound, abdominal palpation and condition of droppings) (22, 23). Animals with unjustifiable suffering equivalent to a scoring of more than 10 points were euthanized. The scoring of all animals was performed by the same investigator.

### Assessment of the Microcirculation

24 h after induction of sepsis, the animals were anesthetized by pentobarbital sodium injection (60 mg • kg ^-1^ body weight i.p.) and buprenorphine (0.05 mg • kg^-1^ s.c.). Animals were placed on a heating pad, tracheotomized and mechanically ventilated in a volume-controlled, pressure-limited mode (70 min ^-1^, VT 1.6 – 2.0 ml, PAW < 17 cm • H_2_O, FiO_2_ = 0.3, Vent Elite, Harvard Apparatus GmbH, March-Hugstetten, Germany). During the experiment 120 µl blood were extracted intermittently (baseline, 45 min, 90 min) for blood gas analysis (BGA) (ABL 800 flex, Radiometer, Copenhagen, Denmark) to check for normocapnic ventilation (p_a_CO_2_ target value 38 ± 5 mmHg), sufficient oxygenation (p_a_O_2_ target value 120 – 150 mmHg) and lactate measurement. If the p_a_CO_2_ or oxygenation target values were not achieved, ventilation or FiO_2_ were adjusted. To ensure continuous anesthesia, an external jugular vein catheter was established for continuous pentobarbital infusion (10 mg • kg^−1^ • h^−1^). Blood pressure and heart rate were measured in the left *arteria carotis communis.* A continuous infusion of crystalloid solution (4 ml • h^-1^) was applied *via* the arterial access for volume replacement and to prevent blood coagulation.

The animals were re-laparotomized and a flexible light guide probe (O_2_C LW 2222, Lea Medizintechnik GmbH, Gießen) was placed on the *tunica serosa* of the colon ascendens, 1 cm distal to the stent and on the left lobe of the rat liver. Microcirculatory oxygenation (µHBO_2_) and perfusion (µflow) were measured as previously described *via* reflectance spectrophotometry and laser Doppler flowmetry (20). White light (450-1000 nm) and laser light (820 nm, 30 mW) were transmitted to the tissue (colonic wall and liver parenchyma) *via* a micro-light guide with a penetration depth of 0.7 mm and the reflected light was analyzed. The wavelength-dependent and overall absorption of the applied white light is used to calculate the percentage of oxygenated hemoglobin in the microcirculation (µHbO_2_). Due to the Doppler effect, magnitude and frequency distribution of changes in wavelength are proportional to the number of blood cells multiplied by the measured mean velocity (µvelo) of these cells. This product is proportional to flow (µflow) and expressed in arbitrary perfusion units (aU). Hence, this method allows the assessment and comparison of oxygenation and perfusion of the examined region at the same time. Only the microcirculation is measured as light entering vessels bigger than 100 µm is completely absorbed. The biggest fraction of the blood volume is stored in venous vessels (85%), so predominantly postcapillary oxygenation respectively mixed liver blood are measured, which represents the critical partial pressure of oxygen (pO_2_) for hypoxia (24). Online evaluation of the signal quality throughout the experiments allows verification of the correct position of the probe tip. The μHbO_2_ and the µflow values reported are the means of the last 5 min every 30 min, beginning at baseline (**Figure 1**).

Thus, 4 different groups of septic animals were analyzed: animals with vehicle-infusion (VE), animals with sodium thiosulfate pretreatment (STS), animals with glibenclamide pretreatment (GL), animals with sodium thiosulfate and glibenclamide pretreatment (STSGL) (**Figure 1**). At the end of the experiments, the animals were euthanized by exsanguination under deep anesthesia and liver and colon tissue samples were harvested.

### Preparation of Liver and Colon Homogenates

Liver and colon homogenates were prepared as described previously (23, 25, 26). Briefly, liver tissue was placed in 4°C cold isolation buffer, minced into 2-3 mm^3^ pieces, rinsed twice in isolation buffer to remove traces of blood and homogenized (Potter-Elvehjem, 5 strokes, 2000 rpm).

Freshly harvested colon was squeezed out to remove faces, then placed in 4°C cold isolation buffer, quickly longitudinally opened and dried softly with a cotton compress. After treatment with trypsin for 5 minutes on ice, tissue was placed in 4°C isolation buffer containing 20 mg/ml BSA and protease inhibitors (*cOmplete*™ Protease Inhibitor Cocktail, Roche Life Science, Mannheim, Germany), minced into 2-3 mm^3^ pieces and homogenized (Potter-Elvehjem, 5 strokes, 2000 rpm).

Protein concentration in the tissue homogenates was determined using the Lowry method with bovine serum albumin as a standard (27).

### Measurement of Mitochondrial Respiratory Rates

For measurement of the mitochondrial oxygen consumption, the oxygen uptake rate was measured at 30°C using a Clark-type electrode (model 782, Strathkelvin instruments, Glasgow, Scotland) as described before (22, 25, 26). Tissue homogenates were suspended in respiration medium (130 mM KCl, 5 mM K_2_HPO_4_, 20 mM MOPS, 2.5 mM EGTA, 1 µM Na_4_P_2_O_7_, 0.1% BSA for liver and 2% BSA for colon, pH 7.15) to yield a protein concentration of 4 mg/ml or 6 mg/ml for liver and colon, respectively.

Mitochondrial state 2 respiration was recorded in the presence of either complex I substrates glutamate and malate (both 2.5 mM, G-M) or complex II substrate succinate (10 mM for liver, 5 mM for colon, S).

The maximal mitochondrial respiration in state 3 was measured after addition of ADP (250 µM for liver, 50 µM for colon). The respiratory control index (RCI) was calculated (state 3/state 2) to define the coupling between the electron transport system and oxidative phosphorylation. To reflect the efficacy of oxidative phosphorylation, the ADP/O ratio was calculated from the amount of ADP added and O_2_-consumption. The average oxygen consumption was calculated as mean from 3 technical replicates.

The solubility of oxygen was assumed to be 223 μmol O_2_ • l^-1^ at 30°C according to the Strathkelvin instruments manual. Respiration rates were expressed as nmol/min/mg protein.

Mitochondria were checked for leakage by addition of 2.5 µM cytochrome c and 0.05 µg/ml oligomycin. Absence of an increase in flux after addition of cytochrome c indicated integrity of the mitochondrial outer membrane. When ATP synthesis was inhibited by oligomycin, the mitochondria were transferred to the state 2, which reflects the respiration rate compensating the proton leak. These results indicate that the inner membrane is intact, and mitochondria were not damaged through the preparation procedure.

### Statistical Analysis

To calculate the appropriate sample size an *a priori* power analysis (G*Power Version 3.1.7, University of Dusseldorf, Germany) was performed. With n = 10 animals per group at a given α ≤ 0.05 (two-tailed) and an expected mean difference in μHbO_2_ of at least 20% (percentage points) with an expected standard deviation of 10 – 15% (based on previous studies) a power of 84.5% resulted.

Normal distribution of data was assessed and confirmed in Q-Q- plots (IBM SPSS Statistics, International Business Machine Corp., Armonk, New York, USA) for microcirculatory data and in Kolmogorov/Smirnov test for mitochondrial results. Microcirculatory data were analyzed with a two-way ANOVA for repeated measures, followed by Dunnett´s post - hoc test for differences versus baseline, and Tukey post-hoc test for differences between groups. For mitochondrial data we used a Kruskal-Wallis test for non-parametric data followed by Dunn’s multiple comparison test (GraphPad software v 6.0, Int., La Jolla, USA). Data are presented as means ± SD for parametric data and as medians, interquartile range (IQR), minimum and maximum for non-parametric data. p < 0.05 was considered significant.

Wherever delta values are presented, the absolute baseline value was subtracted from the absolute value at the respective observation points to individualize the data to each rat’s baseline.

## Results

**Figure 2:**
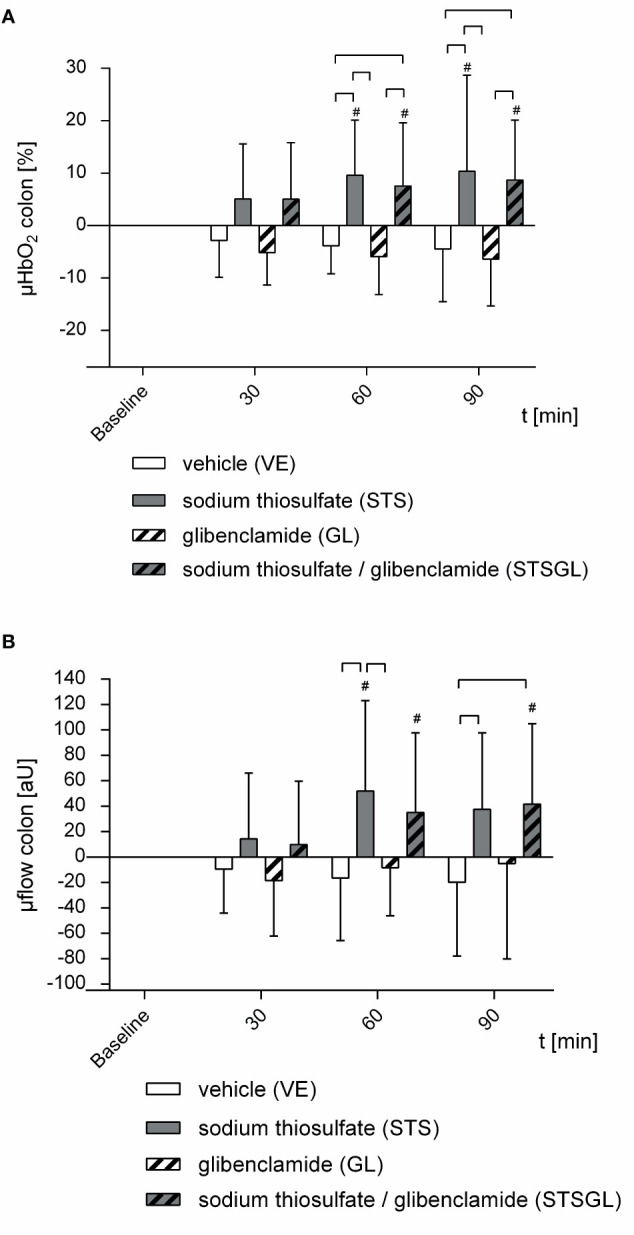
Intestinal microcirculation in septic animals. Effect of sodium thiosulfate (STS), glibenclamide (GL), sodium thiosulfate + glibenclamide (STSGL) or vehicle (VE) on **(A)** colonic microcirculatory oxygenation (µHBO_2_) and **(B)** colonic microcirculatory flow (µflow). Δ µHBO_2_ [%] and Δ µflow [aU] over time calculated to baseline (means ± SD). # = p < 0.05 versus baseline (Two-way ANOVA followed by Dunnett´s *post - hoc* test) _⎴_ between groups (Two-way ANOVA followed by Tukey *post – hoc* test); n = 10.

**Figure 3:**
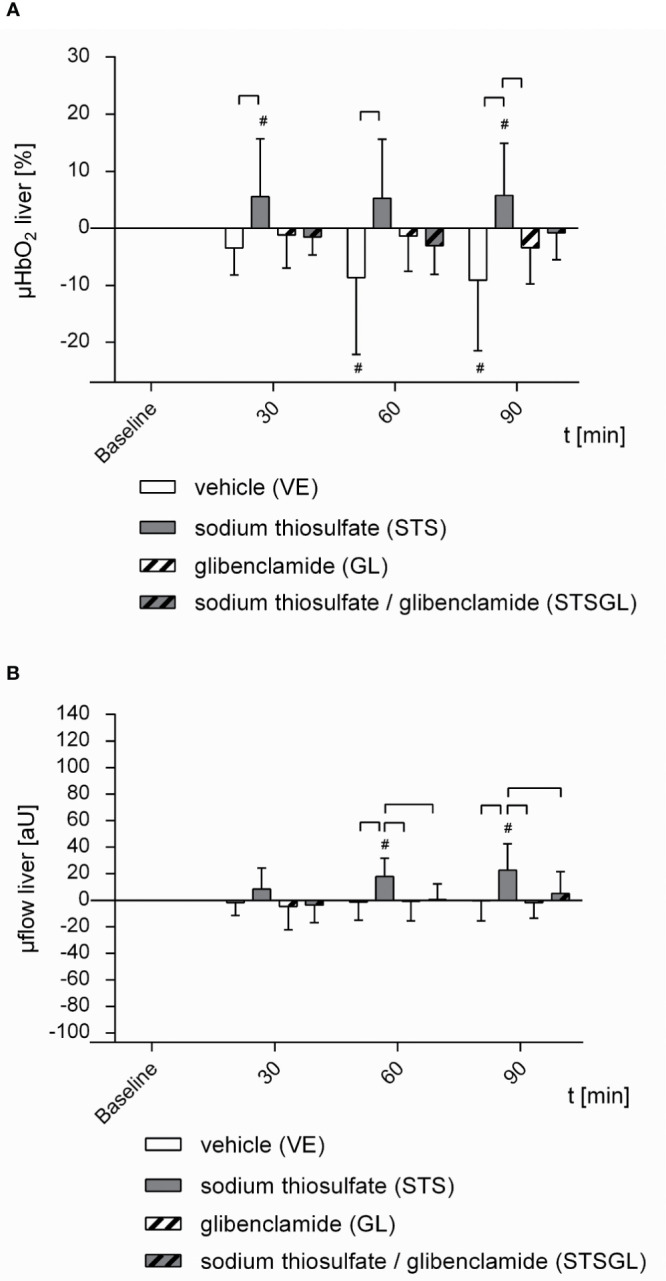
Hepatic microcirculation in septic animals. Effect of sodium thiosulfate (STS), glibenclamide (GL), sodium thiosulfate + glibenclamide (STSGL) or vehicle (VE) on **(A)** hepatic microcirculatory oxygenation (µHBO_2_) and **(B)** hepatic microcirculatory flow (µflow). Δ µHBO_2_ [%] and Δ µflow [aU] over time calculated to baseline (means ± SD). # = p < 0.05 versus baseline (Two-way ANOVA followed by Dunnett´s *post - hoc* test) _⎴_ between groups (Two-way ANOVA followed by Tukey *post – hoc* test); n = 10.

**Figure 4:**
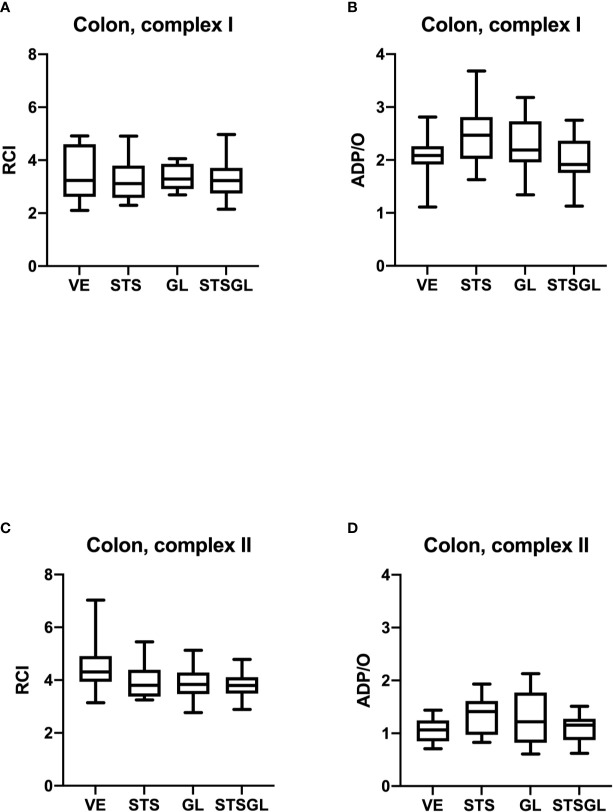
Intestinal mitochondrial function in septic animals. Effect of sodium thiosulfate (STS), glibenclamide (GL) and sodium thiosulfate/glibenclamide (STSGL) compared to control (vehicle: VE) on mitochondrial function of colonic tissue homogenates from septic rats: respiratory control index (RCI) for complex I **(A)** and complex II **(C)** and ADP/ratio for complex I **(B)** and complex II **(D)**. Data are presented as Box-Whisker-Plots presenting median, interquartile range, minimum and maximum value, n=10,.

**Figure 5:**
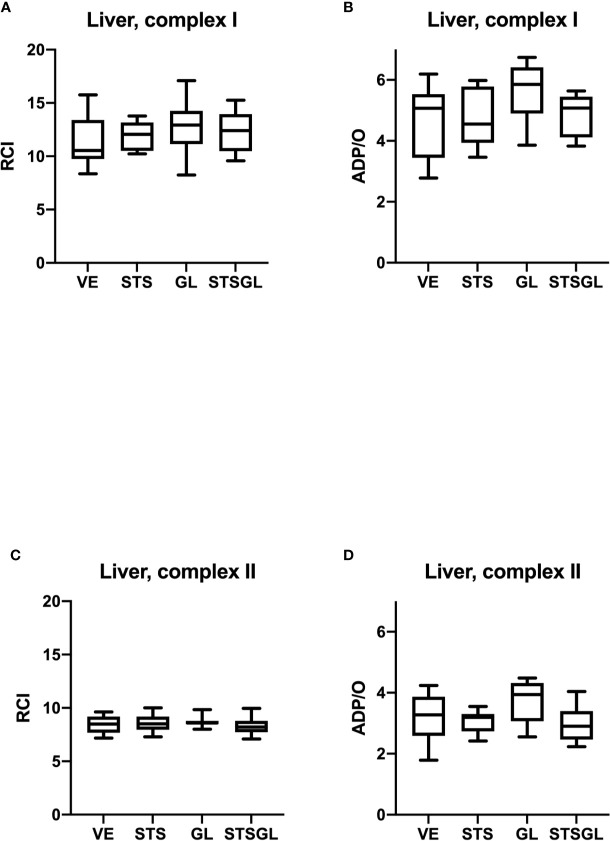
Hepatic mitochondrial function in septic animals. Effect of sodium thiosulfate (STS), glibenclamide (GL) and sodium thiosulfate/glibenclamide(STSGL) compared to control (vehicle: VE) on mitochondrial function of hepatic tissue homogenates from septic rats: respiratory control index (RCI) for complex I **(A)** and complex II **(C)** and ADP/ratio for complex I **(B)** and complex II **(D)**. Data are presented as Box-Whisker-Plots presenting median, interquartile range, minimum and maximum value, n=10,.

**Table 1** and **Figures 2**, **3** summarize the effects of intraperitoneal sodium thiosulfate injection as well as glibenclamide on systemic hemodynamics as well as colonic and liver microcirculation in sepsis. Furthermore, **Figures 4**, **5** summarize the effect on mitochondria.

**Table 1:** Physiological data and SRSS.

		VE	STS	GL	STSGL
lactate [mmol · l^-1^]	Baseline	1.4 ± 0.5	1.6 ± 0.8	1.5 ± 0.3	1.4 ± 0.4
	45 min	1.4 ± 0.3	1.3 ± 0.4	1.3 ± 0.3	1.3 ± 0.4
	90 min	1.4 ± 0.5	1.3 ± 0.6	1.2 ± 0.4	1.2 ± 0.7
					
HR [min^-1^]	Baseline	439 ± 55	425 ± 59	429 ± 33	397 ± 35
	30 min	432 ± 59	426 ± 65	422 ± 34	404 ± 37
	60 min	433 ± 63	424 ± 68	424 ± 47	405 ± 42
	90 min	435 ± 69	425 ± 66	421 ± 53	409 ± 42
					
MAP [mmHg]	Baseline	84 ± 16	81 ± 15	82 ± 13	82 ± 17
	30 min	81 ± 14	85 ± 16	82 ± 12	83 ± 17
	60 min	81 ± 11	84 ± 19	80 ± 14	80 ± 15
	90 min	81 ± 11	84 ± 16	81 ± 15	81 ± 15
					
SRSS	Baseline	4.5 ± 1.1	4.7 ± 1.4	4.4 ± 1.4	4.5 ± 1.3

Lactate levels, heart rate (HR), mean arterial pressure (MAP) and septic rat severity score (SRSS) at baseline. VE, septic animals with vehicle-infusion; STS, septic animals with sodium thiosulfate pretreatment; GL, septic animals with glibenclamide pretreatment; STSGL, septic animals with sodium thiosulfate and glibenclamide. Data are presented as absolute values (means ± SD); n = 10.

24 h after CASP-surgery, baseline values as well as SRSS scores did not differ significantly between the groups (**Table 1**).

### Effect of Intraperitoneal Sodium Thiosulfate on Microcirculation in Septic Animals (STS)

STS increased colonic µHbO_2_ compared to baseline (60 min: + 9.6 ± 10.5%, 90 min: + 10.4 ± 18.3%; p < 0.05 vs. baseline). Colonic µHbO_2_ with STS injection was significantly higher compared to septic animals with vehicle (VE) and glibenclamide (GL) injection (**Figure 2**). Furthermore, sodium thiosulfate ameliorated colonic µflow compared to baseline (60 min: + 51.9 ± 71.1 aU; p < 0.05 vs. baseline) and also compared to septic animals with vehicle (VE) and glibenclamide (GL) injection (**Figure 2**).

Sodium thiosulfate enhanced hepatic µHbO_2_ compared to baseline (30 min: + 5.5 ± 10.1%, 90 min: + 5.8 ± 9.1%; p < 0.05 vs. baseline) as well as compared to septic animals with vehicle (VE) and glibenclamide (GL) injection (**Figure 3**). Hepatic µflow was increased due to sodium thiosulfate compared to baseline (60 min: + 17.8 ± 14.1 aU, 90 min: + 22.5 ± 20.0 aU; p < 0.05 vs. baseline) and significantly increased compared to all other groups (STS vs. VE; GL; STSGL) (**Figure 3**).

MAP, HR and lactate remained unchanged in septic animals after sodium thiosulfate injection. There was also no significant change between groups (**Table 1**).

### Effect of Intraperitoneal Sodium Thiosulfate and Glibenclamide on Microcirculation in Septic Animals (STSGL)

The combination of sodium thiosulfate and glibenclamide increased colonic µHbO_2_ compared to baseline (60 min: + 7.6 ± 12.0%, 90 min: + 8.7 ± 11.5%; p < 0.05 vs. baseline) and µHbO_2_ was significantly higher compared to septic animals with vehicle (VE) and glibenclamide (GL) injection (**Figure 2**). Besides, sodium thiosulfate in combination with glibenclamide increased colonic µflow compared to baseline (60 min: + 35.2 ± 62.6 aU, 90 min: + 41.8 ± 63.3 aU; p < 0.05 vs. baseline) and also compared to septic animals with vehicle (VE) (**Figure 2**).

In contrast to colonic microcirculation, hepatic µHbO and µflow did not change after the combined application of sodium thiosulfate and glibenclamide compared to baseline (**Figure 3**). Likewise, there were no significant changes of hepatic µflow compared to baseline (**Figure 3**).

MAP, HR and lactate remained unchanged in septic animals with sodium thiosulfate and glibenclamide injection. There was also no significant change between groups (**Table 1**).

### Effect of Intraperitoneal Glibenclamide on Microcirculation in Septic Animals (GL)

Glibenclamide did not change colonic and hepatic µHbO_2_ compared to baseline (**Figures 2**, **3**). In addition, there were no significant changes of colonic or hepatic µflow compared to baseline (**Figures 2**, **3**).

MAP, HR and lactate remained unchanged after glibenclamide injection (**Table 1**).

### Effect of Vehicle on Microcirculation in Septic Animals (VE)

In septic animals with vehicle injection there were no significant changes of colonic µHbO_2_ compared to baseline, whereas hepatic µHbO_2_ declined (60 min: - 8.7 ± 13.5%; 90 min: - 9.1 ± 12.3%; p < 0.05 vs. baseline) (**Figures 2**, **3**). Colonic as well as hepatic µflow remained unaltered over the observation period of 90 min (**Figures 2**, **3**).

MAP, HR and lactate remained unchanged in septic animals with vehicle injection (**Table 1**).

### Effect of Intraperitoneal Sodium Thiosulfate and Glibenclamide on Mitochondrial Function in Septic Animals

None of the used substances (applied alone or in combination) affected mitochondrial respiration. There were no statistical differences between control and treated groups regarding RCI and ADP/O ratio after stimulation of the respiratory chain through complex I and II in colonic (**Figure 4**) and hepatic (**Figure 5**) mitochondria.

## Discussion

This study was carried out to evaluate the effect of STS without and with blockade of K(ATP)-channels using glibenclamide on intestinal as well as hepatic microcirculation and mitochondrial function under septic conditions. The main results are:

i) STS ameliorates intestinal microcirculatory oxygenation in septic animals mainly due to improved microcirculatory blood supply. This effect is independent of K(ATP)-channels.
ii) STS also enhances hepatic microcirculatory oxygenation due to improved microcirculatory blood supply. This enhancement seems to be K(ATP)-channel-dependent.
iii) The impact of STS on the intestinal as well as hepatic microcirculation is not mediated by changes in macrohemodynamic variables.
iv) STS does not change mitochondrial function in the intestine and the liver in septic animals.

To investigate the effect of STS and a possible involvement of K(ATP)-channels in septic animals, we used the CASP-model. This well-established experimental sepsis model is one of the closest models to imitate human sepsis with abdominal focus (21). The observed mortality rate of 17% in this study and the SRSS scores are similar to own previous reports and reflect the moderate severity of our sepsis model (20, 21). However, it is of note that animals did not suffer from hypotension or elevated lactate levels as one would expect in septic shock. Thereby, the moderate sepsis model allows to start a therapeutic approach before the development of septic shock and in this way to investigate the early sepsis-induced microcirculatory alterations of the gastrointestinal system (21, 28). Besides, it is of note that an important limitation of the current study is that there is no control group to estimate how severe the sepsis-induced microcirculatory changes are compared to sham operated animals. However, as stated above, the animals showed clear signs of infection: visible peritonitis, SRSS, mortality rate, significantly higher cytokine plasma levels and leucocyte counts compared to sham (as shown in previous studies with the same sepsis model (20, 21). Furthermore, we have already shown in a previous study that induction of a milder sepsis (CASP operation with one 18G-stent) led to a significant deterioration of intestinal microcirculatory oxygenation compared to sham operated animals. Thus, factors such as surgical trauma and related inflammation, impact of the anesthetics etc. can be ruled out (20). Therefore and also to be in accordance with the 3 R tenet of the ARRIVE guidelines we have decided against a control group.

Sepsis induced microcirculatory alterations of the colon and the liver were analyzed *via* reflectance spectrophotometry and laser doppler to simultaneously assess oxygenation and flow (20). This method has been validated in various tissues and is used in different experimental and clinical studies (20, 24, 29–32). Especially under septic conditions reflectance spectrophotometry and laser doppler are valuable analyzing tools, as they measure mainly the postcapillary area and changes in this area correlate with outcome in septic patients (33, 34). It is important to acknowledge that the O_2_C signal averages the whole catchment area and does not provide spatial resolution. This technique is therefore limited in assessment of capillary density or heterogeneity of blood flow. However, an experimental sepsis study could show that amelioration of intestinal oxygenation improves intestinal barrier function and reduces mucosal cell death as indirect outcome parameters, which further underlines the diagnostic relevance of this technique (35).

We decided to assess mitochondrial respiration in tissue homogenates rather than in isolated mitochondria in order to avoid the disadvantages of the isolating procedure (36, 37). Furthermore, to exclude a potential mitochondrial damage due to the preparation procedure, we verified the integrity of the mitochondrial inner and outer membranes.

The dosage of STS used in this study is adapted from the literature (8). Tokuda et al. observed after 2.0 g • kg^-1^ STS i.p. a significantly improved survival rate of septic mice. In their study STS was given after sepsis induction. We applied a dosage of 1.0 g • kg^-1^ STS i.p. at two time points, immediately after sepsis induction and after 24 hours, to include an immediate effect of STS on gastrointestinal microcirculation. Furthermore, we used glibenclamide to investigate if the STS effect is K(ATP)-channel-dependent. Glibenclamide is widely and successfully used as a K(ATP)-channel-blocker in the literature (13, 38–40).

With this study design, we could demonstrate that STS ameliorates intestinal as well as hepatic microcirculatory oxygenation in sepsis. µHbO_2_ in the colon and the liver was significantly higher with STS compared to vehicle treated animals. This improvement of microcirculatory oxygenation seems to be mediated by enhanced microcirculatory perfusion and thereby higher oxygen supply. µflow was also significantly improved in animals treated with STS. Studies concerning the effects of STS on microcirculation are sparse. However, our results are in line with an experimental study of Pihan et al., where STS prevented acute gastrointestinal injury induced by ethanol through maintained microcirculatory flow (41). Furthermore, our results are supported by several studies using H_2_S. As stated above, a possible mechanism of action of STS is the elevation of endogenous H_2_S-levels, especially under ischemic conditions (14, 15). H_2_S improved gastrointestinal microcirculatory perfusion in an ischemia-reperfusion model and furthermore in an experimental sepsis model (7, 42). To date, the microcirculatory mechanism of action of STS, respectively H_2_S, remains unclear. We have demonstrated that improved microcirculation by STS does not seem to be mediated through changes in macrohemodynamic variables. Heart rate and global perfusion pressure remained constant in this study. However, we did not examine further perfusion parameters like cardiac output. However, Volpato et al. demonstrated in their echocardiography study that H_2_S rather leads to reduced cardiac output. Thus, macrohemodynamic changes do not seem to contribute to the observed protective effect of STS (43). Instead, direct local mechanisms of STS and H_2_S might be the reason for increased microcirculatory flow (9, 14, 44).

In this context one main local mechanism of H_2_S seems to be the activation of K(ATP)-channels in the microcirculation inducing vasorelaxation (11, 44). We used glibenclamide, a K(ATP)-channel-blocker, to examine a potential role of activation of K(ATP)-channels. We observed tissue-specific effects. Glibenclamide abolished the protective effect of STS in the hepatic microcirculation. In contrast, the effect of STS in the intestinal microcirculation was unaffected by glibenclamide and thus, seems to be independent of K(ATP)-channel activation.

The results in the hepatic microcirculation are in line with the literature regarding H_2_S. H_2_S induces a vasorelaxant effect in the hepatic vascular bed, predominantly in presinusoidal vessels, *via* K(ATP)-channels, thus, increasing microcirculatory flow (45–47). However, studies relating to the mechanism of action of STS and H_2_S in the intestinal microcirculation are sparse. Regarding our results, K(ATP)-channels do not seem to be a major effector for the amelioration of colonic microcirculatory flow and oxygenation in the context of sepsis. Direct vasodilatory effects of STS and H_2_S *via* eNOS or local receptors like oxytocin receptors might play a role in this context (48, 49). Therefore, further studies are needed to elucidate the exact mechanisms of the microcirculatory effect of STS in the vascular bed of the gastrointestinal tract.

It remains unclear how long the effect of STS in the gastrointestinal microcirculation persists. We observed a profound microcirculatory effect starting at 30 – 60 min after the second injection and lasting until the end of the experiment. Hence, we could show that the effect of STS is lasting for at least 60 min. Furthermore, it remains unclear if the amelioration of gastrointestinal microcirculation due to STS protects against intestinal barrier dysfunction and liver failure in sepsis, as suspected in the literature (35). Therefore, further studies are needed, to investigate the duration of the STS effect and the impact of the microcirculatory changes on gastrointestinal barrier and overall survival in sepsis.

To investigate if the improved microcirculatory oxygenation is only mediated by enhanced microcirculatory flow or if also an optimized mitochondrial function might play a role, we studied colonic and hepatic mitochondrial respiration. Here, we didn’t observe any effect of STS application on colonic and hepatic mitochondrial respiration. Concerning the effect of STS, respectively H_2_S, on mitochondrial function it is known, that low concentrations of H_2_S (0.1 – 1 µM) stimulate mitochondrial oxygen consumption in most organs, while higher concentrations (3 – 30 µM) show an inhibitory effect (50). High sulfide oxidation flux can limit the pool of oxidized coenzyme Q (CoQ), which accepts electrons from complex I and II and inhibit complex IV (51). Unlike many other tissues, the colonic cells are exposed to high levels of H_2_S derived from intestinal microbial metabolism, reaching concentrations of 0.2 – 2.4 mM, depending on the part of the gut (52). In the colon, in *in vitro* experiments with permeabilized cells, concentrations of H_2_S up to 20 µM stimulate mitochondrial respiration, while these concentrations are toxic to other tissues (52, 53). The adaptive mechanisms used by the colonic epithelium to compensate for the exposure to potentially harmful luminal H_2_S concentrations are largely unknown (53). One of the possible mechanisms could be the sulfide oxidation involving the activity of the sulfide quinone reductase (SQR) activity (10).

In our study, we have not observed any effects of STS on mitochondrial respiration. Our results are in line with those of Datzmann et al., who could not show any changes in mitochondrial respiration in brain, heart, kidney or liver after hemorrhagic shock in swine either (54). The impact of hydrogen sulfide on mitochondrial function is known to be reversible. The reversion characteristics like intensity and kinetics are strongly concentration dependent (10). We have not assessed the tissue concentrations of STS and H_2_S. The data from the literature about endogenous tissue concentration of H_2_S are discordant, showing micromolar or nanomolar range and great differences between tissues and species (55–57). Moreover, the accumulation of H_2_S after exogenous application is also highly tissue specific. The increase in H_2_S levels after a subcutaneous application of 60 µg · g^-1^ sodium hydrosulfide in mice accounted for about 50% in kidney and brain, but only 18% in liver (57). If tissue concentrations of H_2_S after intraperitoneal administration of STS are comparable with those after subcutaneous application of sodium hydrosulfide, is not clear. Thus, it remains speculative if in our experimental setting we reached the effective level of H_2_S for affecting the respiratory chain, especially in the colon, where significantly higher concentrations of H_2_S are needed to affect the mitochondrial respiration. Concerning the hepatic mitochondria, another mechanism underlying the lack of changes in mitochondrial respiration is conceivable. We could show that moderate sepsis, as induced by the CASP model, increased mitochondrial respiration in the liver (23). This could depict an adaptative adjustment of the cellular metabolism to preserve the ATP production by impaired oxygen supply under septic conditions. If the adaptive mechanisms are saturated, no further increase in mitochondrial respiration, also under H_2_S, is achievable. Therefore, our results show that the ameliorated microcirculatory oxygenation seems only to be caused by increased oxygen supply and not by changes in mitochondrial respiration.

It is important to note that we studied the role of sodium thiosulfate in a rodent model of sepsis and not in human sepsis, so these data and their clinical impact should be interpreted with care. Nevertheless, our CASP model is one of the closest experimental models to imitate anastomosis failure with consecutive abdominal sepsis. Therefore, our results highlight the potential of sodium thiosulfate in sepsis to protect the intestinal as well as hepatic microcirculation and thus even preventing the vicious circle with intestinal ischemia, translocating bacteria (and toxins) and aggravation of sepsis. However, it is of note that this study has not examined if the enhanced intestinal and hepatic microcirculation due to STS leads to histological improvement, e.g. tight junction performance or cell death. The study focuses on microcirculatory and mitochondrial measurements, based on recent data suggesting that even mild changes in regional oxygenation, which most likely do not lead to visible organ damage have an impact on intestinal barrier function, as shown in a canine model (58). In this setting, i.e. in sepsis before visible tissue damage occurs, there is an opportunity for therapy in the clinical setting, whereas once the tissue is obviously damaged, interventional therapy might be too late (33). Nevertheless, further studies have to be performed to elucidate if STS can also protect organ function in sepsis.

## Conclusion

This randomized, placebo-controlled, blinded animal trial demonstrated that intraperitoneally applied sodium thiosulfate improves intestinal as well as hepatic microcirculatory oxygenation in sepsis. This protective effect seems to be mediated by an increased microcirculatory perfusion and thereby increased oxygen supply and not by changes in mitochondrial respiration or macrohemodynamic variables. We could demonstrate that the intestinal effect of sodium thiosulfate seems to be K(ATP)-channel-independent, whereas the hepatic effect seems to be K(ATP)-channel-dependent. This study reveals a potential new therapy for the compromised gastrointestinal microcirculation during sepsis. Hence, clinical trials have to be performed to investigate the effect of sodium thiosulfate in septic patients.

## Data Availability Statement

The raw data supporting the conclusions of this article will be made available by the authors, without undue reservation.

## Ethics Statement

The animal study was reviewed and approved by Landesamt für Natur, Umwelt und Verbraucherschutz, Recklinghausen, Germany, Az. 84-02.04.2015.A538.

## Author Contributions

JS contributed to the study conception and design, acquisition of data, analysis and interpretation of data and drafting of the article. SK contributed to the acquisition of data, analysis and interpretation of data. YK contributed to the acquisition of data, analysis and interpretation of data. AK contributed to the study conception and design, analysis and interpretation of data and revision of the article. IB contributed to the study conception and design, analysis and interpretation of data and revision of the article. OP contributed to the study conception and design, analysis and interpretation of data and revision of the article. CV contributed to the study conception and design, analysis and interpretation of data and revision of the article. RT contributed to the study conception and design, analysis and interpretation of data and revision of the article. AH contributed to the study conception and design, acquisition of data, analysis and interpretation of data and revision of the article. All authors contributed to the article and approved the submitted version.

## Conflict of Interest

The authors declare that the research was conducted in the absence of any commercial or financial relationships that could be construed as a potential conflict of interest.

## Abbreviations

ATP, Adenosine-triphosphate; ADP, Adenosine-diphospate; ADP/O, Efficacy of oxidative phosphorylation; ANOVA, Analysis of variance; ARRIVE, Animal Research: Reporting In Vivo Experiments; aU, arbitrary perfusion units; BGA, Blood gas analysis; CASP, Colon ascendens stent peritonitis; CoQ, Coenzyme Q; DMSO, Dimethyl sulfoxide; eNOS, endothelial nitric oxide synthase; FiN_2,_ Inspiratory nitrogen concentration; FiO_2,_ Inspiratory oxygen concentration; GL, glibenclamide; H_2_S, Hydrogen sulfide; HR, Heart rate; IQR, inter quartile range; LPS, Lipopolysaccharide; MAP, Mean arterial blood pressure; MODS, Multiorgan dysfunction syndrome; mU, Milli Units; NIH, National Institutes of Health; O_2_C, Oxygen to see; p_a_O_2_, Arterial oxygen partial pressure; p_a_CO_2,_ Arterial carbon dioxide partial pressure; PAW, Airway pressure; PVC, Peripheral venous catheter; RCI, Respiratory control index; s.c., subcutaneous; SD, Standard deviation; SRSS, Septic rat severity score; STS, sodium thiosulfate; SQR, sulfide quinone reductase; VE, vehicle; VT, Tidal volume; µHBO_2,_ Microcirculatory oxygenation; µflow, Microcirculatory flow.

## References

[1] Thompson K Venkatesh B Finfer S. Sepsis and Septic Shock: Current Approaches to Management. Internal Med J (2019) 49(2):160–70. 10.1111/imj.14199 30754087

[2] Otani S Coopersmith CM. Gut Integrity in Critical Illness. J Intensive Care (2019) 7:17. 10.1186/s40560-019-0372-6 30923621 PMC6425574

[3] De Backer D Orbegozo Cortes D Donadello K Vincent J-L. Pathophysiology of Microcirculatory Dysfunction and the Pathogenesis of Septic Shock. Virulence (2014) 5:73–9. 10.4161/viru.26482 PMC3916386 24067428

[4] Sun J Zhang J Wang X Ji F Ronco C Tian J. Gut-Liver Crosstalk in Sepsis-Induced Liver Injury. Crit Care (2020) 24(1):614. 10.1186/s13054-020-03327-1 33076940 PMC7574296

[5] Meng M Klingensmith NJ Coopersmith CM. New Insights Into the Gut as the Driver of Critical Illness and Organ Failure. Curr Opin Crit Care (2017) 23(2):143–8. 10.1097/mcc.0000000000000386 PMC5373099 28092310

[6] Reitsema VA Star BS de Jager VD van Meurs M Henning RH Bouma HR. Metabolic Resuscitation Strategies to Prevent Organ Dysfunction in Sepsis. Antioxidants Redox Signaling (2019) 31(2):134–52. 10.1089/ars.2018.7537 30403161

[7] Pavoni V Nicoletti P Benemei S Materazzi S Perna F Romagnoli S. Effects of Hydrogen Sulfide (H2S) on Mesenteric Perfusion in Experimental Induced Intestinal Ischemia in a Porcine Model. Heart Lung vessels (2015) 7(3):231–7. PMC4593018 26495269

[8] Tokuda K Kida K Marutani E Crimi E Bougaki M Khatri A. Inhaled Hydrogen Sulfide Prevents Endotoxin-Induced Systemic Inflammation and Improves Survival by Altering Sulfide Metabolism in Mice. Antioxidants Redox Signaling (2012) 17(1):11–21. 10.1089/ars.2011.4363 22221071 PMC3342565

[9] Baumgart K Radermacher P Wagner F. Applying Gases for Microcirculatory and Cellular Oxygenation in Sepsis: Effects of Nitric Oxide, Carbon Monoxide, and Hydrogen Sulfide. Curr Opin Anaesthesiol (2009) 22(2):168–76. 10.1097/ACO.0b013e328328d22f 19390245

[10] Leschelle X Goubern M Andriamihaja M Blottiere HM Couplan E Gonzalez-Barroso MD. Adaptative Metabolic Response of Human Colonic Epithelial Cells to the Adverse Effects of the Luminal Compound Sulfide. Biochim Biophys Acta (2005) 1725(2):201–12. 10.1016/j.bbagen.2005.06.002 15996823

[11] Zhao W Zhang J Lu Y Wang R. The Vasorelaxant Effect of H(2)S as a Novel Endogenous Gaseous K(ATP) Channel Opener. EMBO J (2001) 20(21):6008–16. 10.1093/emboj/20.21.6008 PMC125693 11689441

[12] Johansen D Ytrehus K Baxter GF. Exogenous Hydrogen Sulfide (H2S) Protects Against Regional Myocardial Ischemia-Reperfusion Injury–Evidence for a Role of K ATP Channels. Basic Res Cardiol (2006) 101(1):53–60. 10.1007/s00395-005-0569-9 16328106

[13] Spiller F Orrico MI Nascimento DC Czaikoski PG Souto FO Alves-Filho JC. Hydrogen Sulfide Improves Neutrophil Migration and Survival in Sepsis Via K+ATP Channel Activation. Am J Respir Crit Care Med (2010) 182(3):360–8. 10.1164/rccm.200907-1145OC 20339148

[14] Olson KR Deleon ER Gao Y Hurley K Sadauskas V Batz C. Thiosulfate: A Readily Accessible Source of Hydrogen Sulfide in Oxygen Sensing. Am J Physiol Regul Integr Comp Physiol (2013) 305(6):R592–603. 10.1152/ajpregu.00421.2012 23804280

[15] Szabo C Papapetropoulos A. International Union of Basic and Clinical Pharmacology. CII: Pharmacological Modulation of H(2)S Levels: H(2)S Donors and H(2)S Biosynthesis Inhibitors. Pharmacol Rev (2017) 69(4):497–564. 10.1124/pr.117.014050 28978633 PMC5629631

[16] Peng T Zhuo L Wang Y Jun M Li G Wang L. A Systematic Review of Sodium Thiosulfate in Treating Calciphylaxis in Chronic Kidney Disease Patients. Nephrol (Carlton Vic) (2018) 23(7):669–75. 10.1111/nep.13081 28603903

[17] Petrikovics I Budai M Kovacs K Thompson DE. Past, Present and Future of Cyanide Antagonism Research: From the Early Remedies to the Current Therapies. World J Method (2015) 5(2):88–100. 10.5662/wjm.v5.i2.88 PMC4482825 26140275

[18] Brock PR Maibach R Childs M Rajput K Roebuck D Sullivan MJ. Sodium Thiosulfate for Protection From Cisplatin-Induced Hearing Loss. N Engl J Med (2018) 378(25):2376–85. 10.1056/NEJMoa1801109 PMC6117111 29924955

[19] Ravindran S Kurian GA. Preconditioning the Rat Heart With Sodium Thiosulfate Preserved the Mitochondria in Response to Ischemia-Reperfusion Injury. J Bioenergetics Biomembranes (2019) 51(3):189–201. 10.1007/s10863-019-09794-8 30929125

[20] Stübs CCM Picker O Schulz J Obermiller K Barthel F Hahn A-M. Acute, Short-Term Hypercapnia Improves Microvascular Oxygenation of the Colon in an Animal Model of Sepsis. Microvascular Res (2013) 90:180–6. 10.1016/j.mvr.2013.07.008 23916914

[21] Schulz J Schoneborn S Vollmer C Truse R Herminghaus A Bauer I. Hypercapnia-Induced Amelioration of the Intestinal Microvascular Oxygenation in Sepsis Is Independent of the Endogenous Sympathetic Nervous System. Shock (2018) 49(3):326–33. 10.1097/shk.0000000000000920 28650926

[22] Herminghaus A Barthel F Heinen A Beck C Vollmer C Bauer I. Severity of Polymicrobial Sepsis Modulates Mitochondrial Function in Rat Liver. Mitochondrion (2015) 24:122–8. 10.1016/j.mito.2015.08.001 26277734

[23] Herminghaus A Papenbrock H Eberhardt R Vollmer C Truse R Schulz J. Time-Related Changes in Hepatic and Colonic Mitochondrial Oxygen Consumption After Abdominal Infection in Rats. Intensive Care Med Exp (2019) 7(1):4. 10.1186/s40635-018-0219-9 30623256 PMC6325055

[24] Siegemund M van Bommel J Ince C. Assessment of Regional Tissue Oxygenation. Intensive Care Med (1999) 25(10):1044–60. 10.1007/s001340051011 10551958

[25] Herminghaus A Buitenhuis AJ Schulz J Vollmer C Scheeren TWL Bauer I. Propofol Improves Colonic But Impairs Hepatic Mitochondrial Function in Tissue Homogenates From Healthy Rats. Eur J Pharmacol (2019) 853:364–70. 10.1016/j.ejphar.2019.04.031 31009637

[26] Herminghaus A Laser E Schulz J Truse R Vollmer C Bauer I. Pravastatin and Gemfibrozil Modulate Differently Hepatic and Colonic Mitochondrial Respiration in Tissue Homogenates From Healthy Rats. Cells (2019) 8(9):983. 10.3390/cells8090983 PMC6769625 31461874

[27] Lowry OH Rosebrough NJ Farr AL Randall RJ. Protein Measurement With the Folin Phenol Reagent. J Biol Chem (1951) 193(1):265–75. 14907713

[28] Lustig MK Bac VH Pavlovic D Maier S Gründling M Grisk O. Colon Ascendens Stent Peritonitis–A Model of Sepsis Adopted to the Rat: Physiological, Microcirculatory and Laboratory Changes. Shock (Augusta Ga) (2007) 28:59–64. 10.1097/SHK.0b013e31802e454f 17483746

[29] Bludau M Vallbohmer D Gutschow C Holscher AH Schroder W. Quantitative Measurement of Gastric Mucosal Microcirculation Using a Combined Laser Doppler Flowmeter and Spectrophotometer. Dis Esophagus (2008) 21(7):668–72. 10.1111/j.1442-2050.2008.00856.x 18564159

[30] Sturm T Leiblein J Schneider-Lindner V Kirschning T Thiel M. Association of Microcirculation, Macrocirculation, and Severity of Illness in Septic Shock: A Prospective Observational Study to Identify Microcirculatory Targets Potentially Suitable for Guidance of Hemodynamic Therapy. J Intensive Care Med (2018) 33(4):256–66. 10.1177/0885066616671689 27686326

[31] Klein KU Schramm P Glaser M Reisch R Tresch A Werner C. Intraoperative Monitoring of Cerebral Microcirculation and Oxygenation–A Feasibility Study Using a Novel Photo-Spectrometric Laser-Doppler Flowmetry. J Neurosurg Anesthesiol (2010) 22(1):38–45. 10.1097/ANA.0b013e3181bea439 19816204

[32] Forst T Hohberg C Tarakci E Forst S Kann P Pfutzner A. Reliability of Lightguide Spectrophotometry (O2C) for the Investigation of Skin Tissue Microvascular Blood Flow and Tissue Oxygen Supply in Diabetic and Nondiabetic Subjects. J Diabetes Sci Technol (2008) 2(6):1151–6. 10.1177/193229680800200625 PMC2769807 19885305

[33] De Backer D Donadello K Sakr Y Ospina-Tascon G Salgado D Scolletta S. Microcirculatory Alterations in Patients With Severe Sepsis: Impact of Time of Assessment and Relationship With Outcome. Crit Care Med (2013) 41:791–9. 10.1097/CCM.0b013e3182742e8b 23318492

[34] Mayer K Trzeciak S Puri NK. Assessment of the Adequacy of Oxygen Delivery. Curr Opin Crit Care (2016) 22(5):437–43. 10.1097/mcc.0000000000000336 27467272

[35] Yeh YC Wu CY Cheng YJ Liu CM Hsiao JK Chan WS. Effects of Dexmedetomidine on Intestinal Microcirculation and Intestinal Epithelial Barrier in Endotoxemic Rats. Anesthesiology (2016) 125(2):355–67. 10.1097/aln.0000000000001135 27111533

[36] Pecinová A Drahota Z Nůsková H Pecina P Houštěk J. Evaluation of Basic Mitochondrial Functions Using Rat Tissue Homogenates. Mitochondrion (2011) 11(5):722–8. 10.1016/j.mito.2011.05.006 21664301

[37] Kozlov AV Duvigneau JC Hyatt TC Raju R Behling T Hartl RT. Effect of Estrogen on Mitochondrial Function and Intracellular Stress Markers in Rat Liver and Kidney Following Trauma-Hemorrhagic Shock and Prolonged Hypotension. Mol Med (Cambridge Mass) (2010) 16(7-8):254–61. 10.2119/molmed.2009.00184 PMC2896467 20379612

[38] Beck C Barthel F Hahn A-M Vollmer C Herminghaus A Schäfer S. The Beneficial Effects of Acute Hypercapnia on Microcirculatory Oxygenation in an Animal Model of Sepsis are Independent of K(+)ATP Channels. Microvascular Res (2015) 99:78–85. 10.1016/j.mvr.2015.02.009 25758765

[39] Hajhashemi V Amin B. Effect of Glibenclamide on Antinociceptive Effects of Antidepressants of Different Classes. Clinics (Sao Paulo) (2011) 66(2):321–5. 10.1590/s1807-59322011000200023 PMC3059867 21484053

[40] Iskit AB Erkent U Ertunc M Guc MO Ilhan M Onur R. Glibenclamide Attenuates the Antiarrhythmic Effect of Endotoxin With a Mechanism Not Involving K(ATP) Channels. Vasc Pharmacol (2007) 46(2):129–36. 10.1016/j.vph.2006.08.415 17064967

[41] Pihan G Majzoubi D Haudenschild C Trier JS Szabo S. Early Microcirculatory Stasis in Acute Gastric Mucosal Injury in the Rat and Prevention by 16,16-Dimethyl Prostaglandin E2 or Sodium Thiosulfate. Gastroenterology (1986) 91(6):1415–26. 10.1016/0016-5085(86)90195-2 2945748

[42] Ahmad A Druzhyna N Szabo C. Delayed Treatment With Sodium Hydrosulfide Improves Regional Blood Flow and Alleviates Cecal Ligation and Puncture (Clp)-Induced Septic Shock. Shock (2016) 46(2):183–93. 10.1097/shk.0000000000000589 PMC4949092 26863032

[43] Volpato GP Searles R Yu B Scherrer-Crosbie M Bloch KD Ichinose F. Inhaled Hydrogen Sulfide: A Rapidly Reversible Inhibitor of Cardiac and Metabolic Function in the Mouse. Anesthesiology (2008) 108(4):659–68. 10.1097/ALN.0b013e318167af0d PMC2838418 18362598

[44] Guettler C Kubes P. Hydrogen Sulfide, Another Simple Gas With Complex Biology. Am J Physiol Gastrointestinal Liver Physiol (2013) 304(12):G1066–9. 10.1152/ajpgi.00125.2013 23639806

[45] Fiorucci S Antonelli E Mencarelli A Orlandi S Renga B Rizzo G. The Third Gas: H2S Regulates Perfusion Pressure in Both the Isolated and Perfused Normal Rat Liver and in Cirrhosis. Hepatology (Baltimore Md) (2005) 42(3):539–48. 10.1002/hep.20817 16108046

[46] Distrutti E Mencarelli A Santucci L Renga B Orlandi S Donini A. The Methionine Connection: Homocysteine and Hydrogen Sulfide Exert Opposite Effects on Hepatic Microcirculation in Rats. Hepatology (Baltimore Md) (2008) 47(2):659–67. 10.1002/hep.22037 18098324

[47] Norris EJ Larion S Culberson CR Clemens MG. Hydrogen Sulfide Differentially Affects the Hepatic Vasculature in Response to Phenylephrine and Endothelin 1 During Endotoxemia. Shock (2013) 39(2):168–75. 10.1097/SHK.0b013e3182736688 23143058

[48] Denoix N McCook O Ecker S Wang R Waller C Radermacher P. The Interaction of the Endogenous Hydrogen Sulfide and Oxytocin Systems in Fluid Regulation and the Cardiovascular System. Antioxidants (Basel Switzerland) (2020) 9(8):748. 10.3390/antiox9080748 PMC7465147 32823845

[49] Szabo C. Hydrogen Sulfide, an Enhancer of Vascular Nitric Oxide Signaling: Mechanisms and Implications. Am J Physiol Cell Physiol (2017) 312(1):C3–c15. 10.1152/ajpcell.00282.2016 27784679 PMC5283895

[50] Módis K Coletta C Erdélyi K Papapetropoulos A Szabo C. Intramitochondrial Hydrogen Sulfide Production by 3-Mercaptopyruvate Sulfurtransferase Maintains Mitochondrial Electron Flow and Supports Cellular Bioenergetics. FASEB J (2013) 27(2):601–11. 10.1096/fj.12-216507 23104984

[51] Nicholls P Kim JK. Sulphide as an Inhibitor and Electron Donor for the Cytochrome C Oxidase System. Can J Biochem (1982) 60(6):613–23. 10.1139/o82-076 6288202

[52] Macfarlane GT Gibson GR Cummings JH. Comparison of Fermentation Reactions in Different Regions of the Human Colon. J Appl bacteriology (1992) 72(1):57–64. 10.1111/j.1365-2672.1992.tb04882.x 1541601

[53] Libiad M Vitvitsky V Bostelaar T Bak DW Lee HJ Sakamoto N. Hydrogen Sulfide Perturbs Mitochondrial Bioenergetics and Triggers Metabolic Reprogramming in Colon Cells. J Biol Chem (2019) 294(32):12077–90. 10.1074/jbc.RA119.009442 PMC6690701 31213529

[54] Datzmann T Hoffmann A McCook O Merz T Wachter U Preuss J. Effects of Sodium Thiosulfate (Na(2)s(2)O(3)) During Resuscitation From Hemorrhagic Shock in Swine With Preexisting Atherosclerosis. Pharmacol Res (2020) 151:104536. 10.1016/j.phrs.2019.104536 31734346

[55] Ogasawara Y Isoda S Tanabe S. Tissue and Subcellular Distribution of Bound and Acid-Labile Sulfur, and the Enzymic Capacity for Sulfide Production in the Rat. Biol Pharm Bull (1994) 17(12):1535–42. 10.1248/bpb.17.1535 7735193

[56] Furne J Saeed A Levitt MD. Whole Tissue Hydrogen Sulfide Concentrations are Orders of Magnitude Lower Than Presently Accepted Values. Am J Physiol Regul Integr Comp Physiol (2008) 295(5):R1479–85. 10.1152/ajpregu.90566.2008 18799635

[57] Mitchell TW Savage JC Gould DH. High-Performance Liquid Chromatography Detection of Sulfide in Tissues From Sulfide-Treated Mice. J Appl Toxicol JAT (1993) 13(6):389–94. 10.1002/jat.2550130605 8288842

[58] Truse R Hinterberg J Schulz J Herminghaus A Weber A Mettler-Altmann T. Effect of Topical Iloprost and Nitroglycerin on Gastric Microcirculation and Barrier Function During Hemorrhagic Shock in Dogs. J Vasc Res (2017) 54(2):109–21. 10.1159/000464262 28441653

